# Current clinical applications of coronary optical coherence tomography

**DOI:** 10.1007/s12928-017-0483-8

**Published:** 2017-07-14

**Authors:** Teruyoshi Kume, Shiro Uemura

**Affiliations:** Department of Cardiology, Kawasaki Medical School, 577 Matsushima, Kurashiki, Okayama 701-0192 Japan

**Keywords:** Optical coherence tomography, Imaging, Vulnerable plaque, Thrombus, Coronary intervention

## Abstract

Optical coherence tomography (OCT) is an intra-coronary diagnostic technique that provides detailed imagings of blood vessels in the current cardiac catheterization laboratory. The higher resolution of OCT often provides superior delineation of each structure compared with intravascular ultrasound (IVUS), and it can reliably visualize the microstructure of normal and diseased arteries. The capabilities of OCT are well suited for the identification of calcified plaque and neointima formation after stent implantation. It has been reported that OCT-guided percutaneous coronary intervention (PCI) resulted in equivalent clinical and angiographic outcomes in comparison with IVUS-guided PCI. Recently, the three-dimensional reconstruction of OCT and a real-time point-to-point correspondence between coronary angiographic and OCT/OFDI images have been developed and provide useful information to PCI operators. The unique capabilities of OCT as an investigational tool for high-risk lesions will serve the cardiology community well, as it moves us toward a better understanding of atherosclerotic plaque. In addition, because of the development of new OCT technology, OCT has become a notable catheter-based imaging technology that can provide practical guidance for PCI in clinical settings.

## Introduction

Optical coherence tomography (OCT) is an intra-coronary diagnostic technique that provides detailed imagings of blood vessels in the current cardiac catheterization laboratory. The first OCT system was developed by a group of James G. Fujimoto in 1991 [1]. By the early 2000s, the first images of human coronary atherosclerosis were recorded by Yabushita and colleagues [2]. In 2008, the first commercially available time-domain OCT system (M2 OCT imaging system, LightLab Imaging, Inc., Westford, MA, USA) was introduced and included under insurance coverage in Japan. However, the time-domain OCT system needed an over-the-wire-type catheter with an occlusion balloon to obtain continuous long-sectional images due to the limitation of pullback speed. Therefore, the application of time-domain OCT was mostly limited to research purposes. More recently, new generation OCT systems, such as frequency-domain OCT and optical frequency-domain imaging (OFDI) systems, have been developed to overcome this limitation [3]. Since then, OCT has become a noticeable catheter-based imaging technology that can provide scientific insights into vascular biology and practical guidance for percutaneous intervention (PCI) in clinical settings. In the current review article, updates on OCT image interpretation and clinical applications of coronary OCT are discussed.

## OCT image interpretation

### Coronary artery morphology

The higher resolution of OCT often provides superior delineation of each structure compared with intravascular ultrasound (IVUS), and it can reliably visualize the microstructure (i.e., 15–50 vs. 150–200 µm for IVUS) of normal and diseased arteries. Typically, the media of the vessel appear as a lower signal intensity band than the intima and adventitia, providing a three-layered appearance. There is good agreement in the intimal thickness between OCT and histological examination (*r* = 0.98, *p* < 0.001, mean difference = 0.01 ± 0.04 mm) [4]. Most adults undergoing cardiac catheterization have intimal thickening and a signal-rich thick intimal band, even in angiographically normal segments. OCT signal penetration through the diseased arterial wall is generally more limited (no more than 2 mm with current OCT devices), making it difficult to investigate deeper portions of the artery or to track the entire circumference of the media–adventitia interface.

### Plaque characterization

**Fig. 1:**
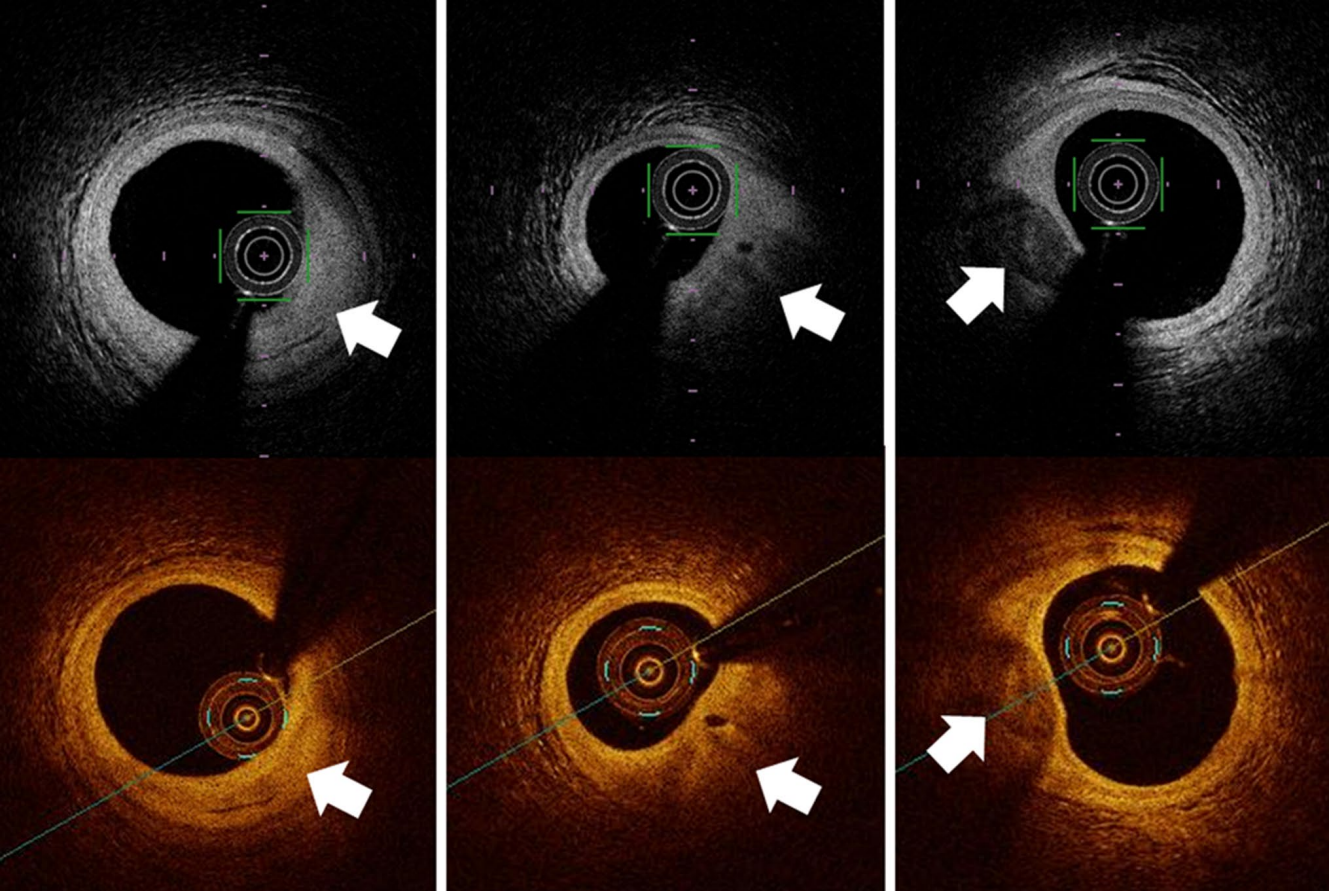
Plaque characterization of OCT images (optical frequency-domain imaging systems: *top*, and frequency-domain OCT: *bottom*). Fibrous plaques exhibit homogeneous, signal-rich (highly backscattering) regions (*left*, *arrow*); lipid-rich plaques exhibit signal-poor regions with poorly defined borders (*middle*, *arrow*); and calcified plaques exhibit signal-poor regions with sharply delineated upper and/or lower borders (*right*, *arrow*)

Fibrous plaques exhibit homogeneous, signal-rich (highly backscattering) regions; lipid-rich plaques exhibit signal-poor regions (lipid pools) with poorly defined borders; and calcified plaques exhibit signal-poor regions with sharply delineated upper and/or lower borders (Fig. 1). OCT has the advantage of being able to image through calcium without shadowing, as would be seen with IVUS. Therefore, there is a better correlation in areas of calcification between OCT and histological examination than between IVUS and the histological examination (*r* = 084, *p* < 0.001 vs. *r* = 0.78, *p* < 0.001) [5]. These data suggest that superficial calcification can be quantified more accurately using OCT than with IVUS.

### Detection of vulnerable plaque

One of the most valuable challenges for OCT is its role in the detection of vulnerable plaque. Myocardial infarction, sudden cardiac death, and unstable angina arise from coronary thrombosis, which mainly develops as a result of ruptured vulnerable plaque. Autopsy studies have identified several histological characteristics of plaques that correlate with the risk of rupture and subsequent acute coronary events. These characteristics include: (1) a large necrotic core with an overlying thin fibrous cap (<65 µm), so-called thin-cap fibroatheroma (TCFA); (2) activated macrophages near the fibrous cap; and (3) neoangiogenesis [6]. A TCFA is characterized as a necrotic core with an overlying fibrous cap. A fibrous cap consists of vascular smooth muscle cell and extracellular matrix, which is often a signal-rich band, overlying a signal-poor region indicating the necrotic core. In the OCT image, the necrotic core is characterized as signal-poor regions with poorly defined borders, similar to that of a lipid pool. Histologically, a lipid pool is converted to a necrotic core by macrophage infiltration and apoptosis forming the early necrotic core. In previous studies by a combination of pathohistology and OCT, researchers did not attempt to differentiate a necrotic core from a lipid pool, although these structures have different clinical relevance [2, 7]. Recently, Fujii and colleagues reported that the histological findings underlying the false-positive diagnoses of OCT for TCFA included foam cell accumulation on the luminal surface, microcalcifications at the surface, hemosiderin accumulation, or organized thrombus [8]. It seems likely that the diagnostic accuracy for OCT-derived TCFA was not higher than expected, so the ability of OCT to characterize a lipid pool containing necrotic core needs to be clarified in future histologic studies.

**Fig. 2:**
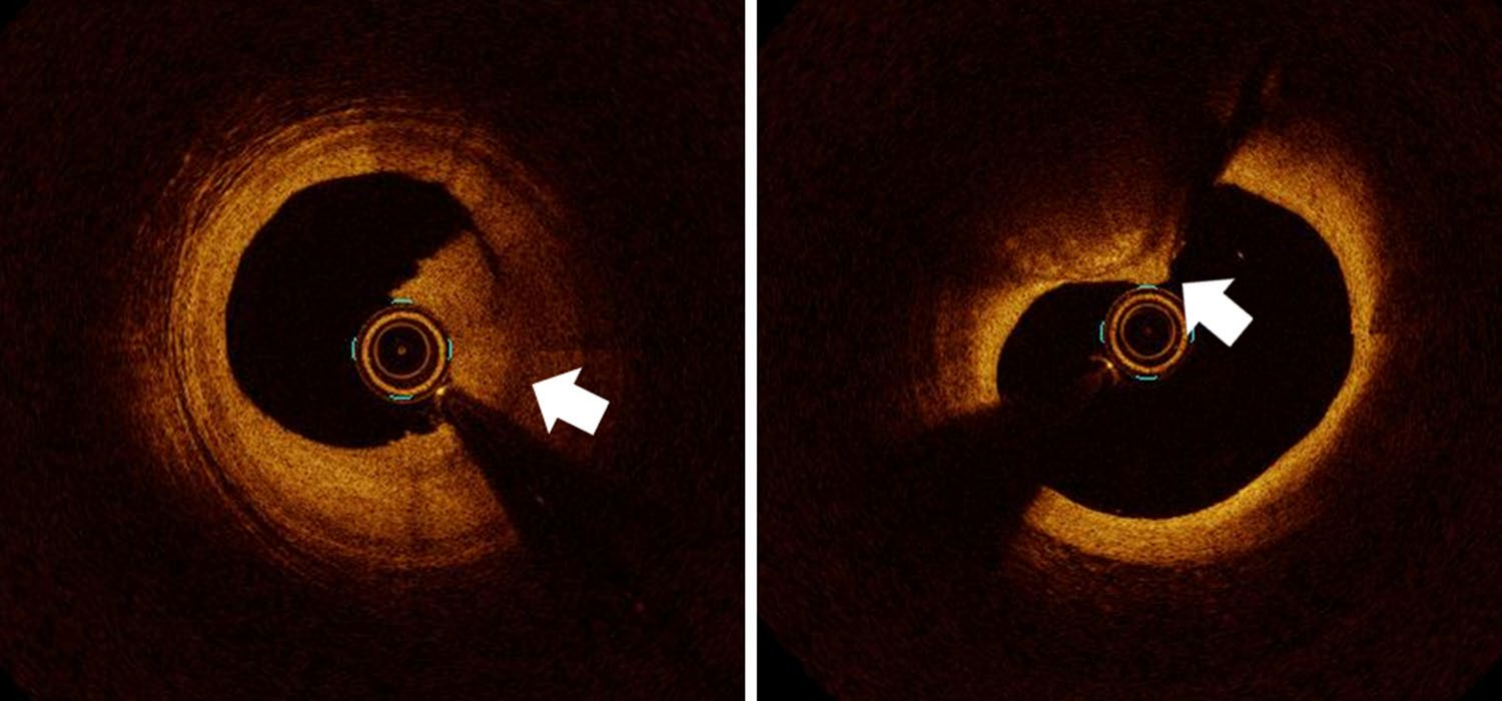
Plaque erosion and calcified nodule. Plaque erosion defined by OCT is characterized as intact fibrous cap with thrombosis and underlying plaque structure can be visualized (*left*, *arrow*). OCT-defined calcified nodule is characterized when fibrous cap disruption was detected over a protruding calcification, superficial calcium, and the presence of substantive calcium proximal, and/or distal to the lesion (*right*, *arrow*)

Plaque neoangiogenesis is another characteristic of vulnerable plaque. Newly formed angiogenic vessels are fragile and tend to break. This may result in an intraplaque hemorrhage, eventually resulting in luminal narrowing [9]. In cross-sectional OCT images, neoangiogenesis can be recognized as small black holes or tubes (microchannels) [10]. Microchannels detected by OCT are associated with plaque vulnerability and are predictors of luminal narrowing in clinical studies [11, 12]. In our ex vivo study using a time-domain OCT system (first-generation OCT system), OCT examination of neovascularization in coronary plaque was feasible, but it had low sensitivity for the detection of neovascularization [13]. The accuracy of currently available OCT systems for the detection of coronary plaque neoangiogenesis has not yet been investigated. Other vulnerable plaque characteristics that may cause acute coronary syndrome are plaque erosion and calcified nodules (Fig. 2) [14]. Plaque erosions are responsible for one-third of patients experiencing sudden cardiac death [6]. Pathologically, plaque erosion is described as the intact fibrous cap with superficial endothelial cells dysfunctional and insufficient. Current OCT system is not sensitive enough to detect endothelial cells insufficient directly. Plaque erosion defined by OCT (OCT-erosion) is characterized as intact fibrous cap with thrombosis and underlying plaque structure can be visualized [14]. Probable OCT erosion includes two types: first, intact fibrous cap with no thrombosis in and around the culprit lesion, and luminal surface is irregular and second, there is thrombosis at the culprit lesion site resulting in the invisibility of underlying plaque structure, and superficial lipid pool and calcification will not be found proximally or distally to the thrombus. Calcified nodule defined by OCT (OCT-calcified nodule) is characterized when fibrous cap disruption was detected over a protruding calcification, superficial calcium, and the presence of substantive calcium proximal and/or distal to the lesion.

The unique capabilities of OCT as an investigational tool for high-risk lesions will serve the cardiology community well, as it moves us toward a better understanding and identification of vulnerable plaque, thereby improving our ability to more precisely treat our patients, both acutely and for the long term.

### Thrombus evaluation

**Fig. 3:**
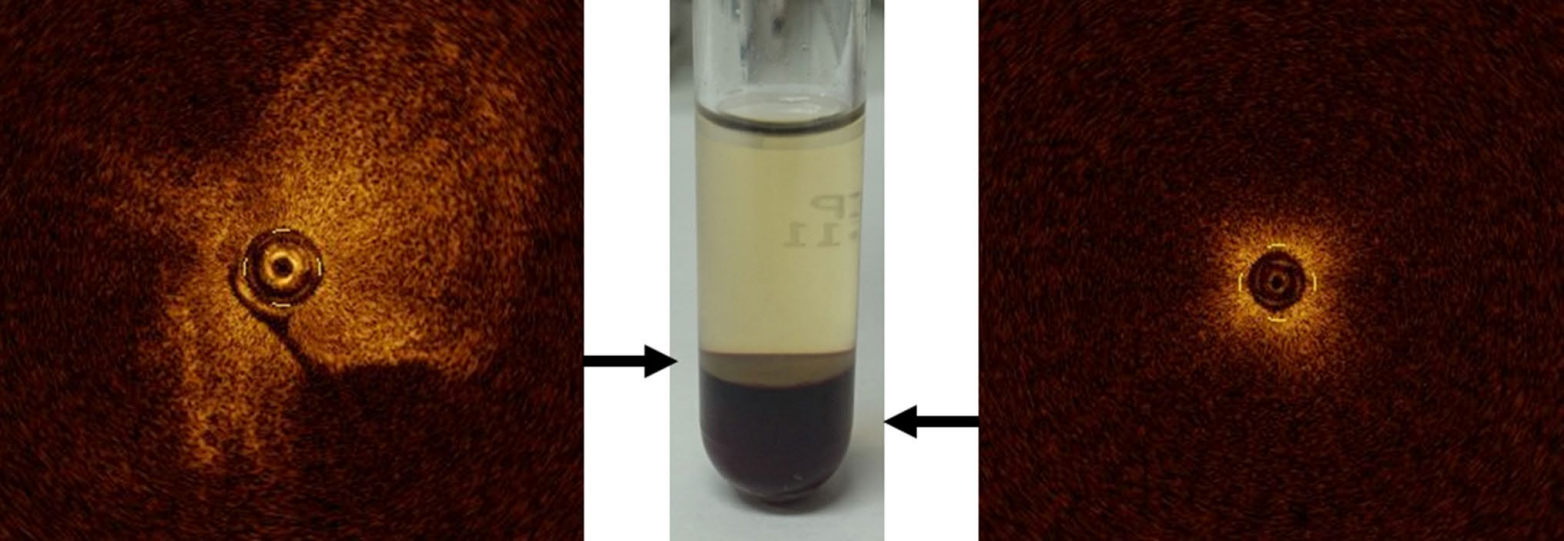
Red blood cells, white blood cells, and platelets separated by centrifugation and corresponding OCT images. Centrifuged whole blood showing plasma, the buffy coat containing white blood cells and platelets, and red blood cells. The buffy coat is characterized by a homogeneous signal that is rich with low backscattering on OCT imaging (*left*). On the other hand, the red blood cell layer is identified as a highly backscattering and highly attenuated light signal resembling blood (*right*)

Most acute coronary events, including myocardial infarction, unstable angina, and sudden cardiac death, occur in relation with subsequent coronary thrombus formation based on three pathological conditions in the coronary arterial wall: ruptured vulnerable plaque, plaque erosion, or a denuded calcified nodule [14]. Thrombi that form within regions of blood stasis or low flow are composed of red blood cells entrapped within interspersed fibrin and a smaller component of aggregated platelets. These thrombi are colored darkly by abundant hemoglobin and are often referred to as “red” thrombi. In contrast, platelet-rich thrombi, which contain few red cells, appear off-white in color and are called “white” thrombi. Red thrombus is depicted as a high-backscattering protrusion with signal-free shadowing in OCT images. White thrombus is depicted by a signal-rich, low-backscattering projection. There is a significant difference in attenuation of intensity between red and white thrombi [15]. OCT utilizes low coherence sources that emit a very broad bandwidth (range of wavelengths within the beam) of near infrared light. Theoretically, red blood cells significantly scatter the emitted light at this wavelength, and the degree of attenuation through red blood cells is greater than that through other blood components, such as white blood cells or platelets. As shown in Fig. 3, centrifuged whole blood consists of three layers: plasma (top), buffy coat (middle), and red blood cells (bottom). The middle thin buffy coat layer mainly contains white blood cells and platelets, and it is characterized by a homogeneous signal that is rich with low backscattering in the OCT image. On the other hand, the dark red bottom layer contains red blood cells and is identified as a highly backscattering and highly attenuated light signal. This simple experiment clearly demonstrates that OCT is able to discriminate platelet-rich thrombus from red blood cell-rich thrombus. Impact of characterization of thrombi is still unclear in the clinical situation. Further study is needed to clarify the clinical impact of discriminating platelet-rich thrombus from red blood cell-rich thrombus in the future.

**Fig. 4:**
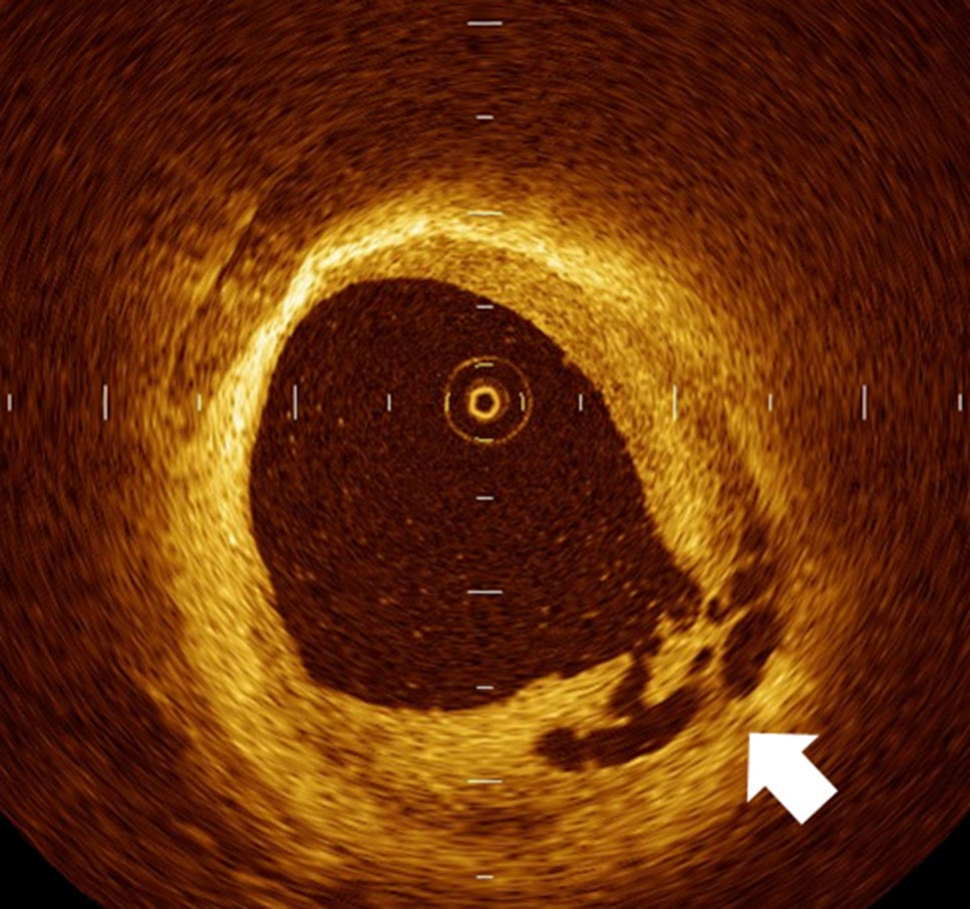
Recanalized thrombus in an OCT image. Recanalized thrombi show signal-rich, high backscattered septae that divide the lumen into multiple small cavities. These cavities communicate with each other on OCT images (*arrow*). The thin septae have a “Swiss cheese” appearance

Older coronary thrombi undergo organization with recanalization to form multiple de novo lumens with various sizes. One-third of patients with thrombotic occlusion were reported to have some degree of recanalization, as confirmed by pathological examination [16]. Although the OCT characteristics of old thrombus are not well validated, one OCT study by Kang and colleagues described the appearance of recanalized thrombus on OCT images [17]. Recanalized thrombi showed signal-rich, high backscattered septae that divided the lumen into multiple small cavities. These cavities communicated with each other on OCT images (Fig. 4). The thin septae had a “Swiss cheese” appearance. This finding is also known as a “lotus root” appearance on IVUS images [18].

### Assessment of neointima

The capabilities of OCT are well suited for the identification of neointima formation, where the relevant morphologic features are primarily localized within 500 μm of the luminal surface. Previous ex vivo studies have reported that OCT can characterize neointimal tissue morphologies following coronary stent implantation [19–21]. Homogeneous neointima including predominantly smooth muscle cells with collagen fibers is typically observed after bare-metal stent implantation, and it has been reported that homogeneous neointima is a predictor of late neointimal regression during 18 month follow-up [22]. On the other hand, heterogeneous neointima contains various tissue components, such as proteoglycan-rich myxomatous matrix, calcium deposition, foam cell accumulation, and fibroatheroma. Heterogeneous neointima with high attenuation that cause invisible stent struts indicates a large amount of foam cell accumulation and large fibroatheroma/necrotic core within neointima. Such heterogeneous neointima, so-called neo-atherosclerosis, is a frequent finding in lesions with drug-eluting stent implantation and observed earlier than in bare-metal stent implantation [23]. Although the mechanism of very late stent thrombosis is multifactorial [24], rupture of an atheromatous lesion within the stented segment (neointimal rupture) may be associated with the occurrence of very late stent thrombosis. OCT is useful for monitoring the morphological changes that occur in neointima formation after stent implantation. In addition, when various types of PCI, including plain old balloon angioplasty, paclitaxel-coated balloon dilatation, and drug-eluting stent implantation, for in-stent restenosis (ISR) lesions were compared, target lesion revascularization (TLR) rates of lesions with a homogeneous neointima were significantly higher in the plain old balloon angioplasty group than in the paclitaxel-coated balloon dilatation group (ISR: 54.8 vs. 19.1%, *p* < 0.001; TLR: 38.7 vs. 10.6%, *p* < 0.001) and drug-eluting stent group (ISR: 54.8 vs. 19.6%, *p* = 0.002; TLR: 38.7 vs. 10.7%, *p* = 0.005), whereas there were no differences among the three groups in lesions with a heterogeneous structure [25]. These results suggested that treatment using a paclitaxel-coated balloon or drug-eluting stent group might be preferable for ISR lesions with homogeneous neointima compared with plain old balloon angioplasty. Morphological assessment of neointima in lesions with ISR using OCT provides useful information about suitable PCI strategies for ISR lesions.

## Interventional applications

### Guidance for coronary interventions

**Fig. 5:**
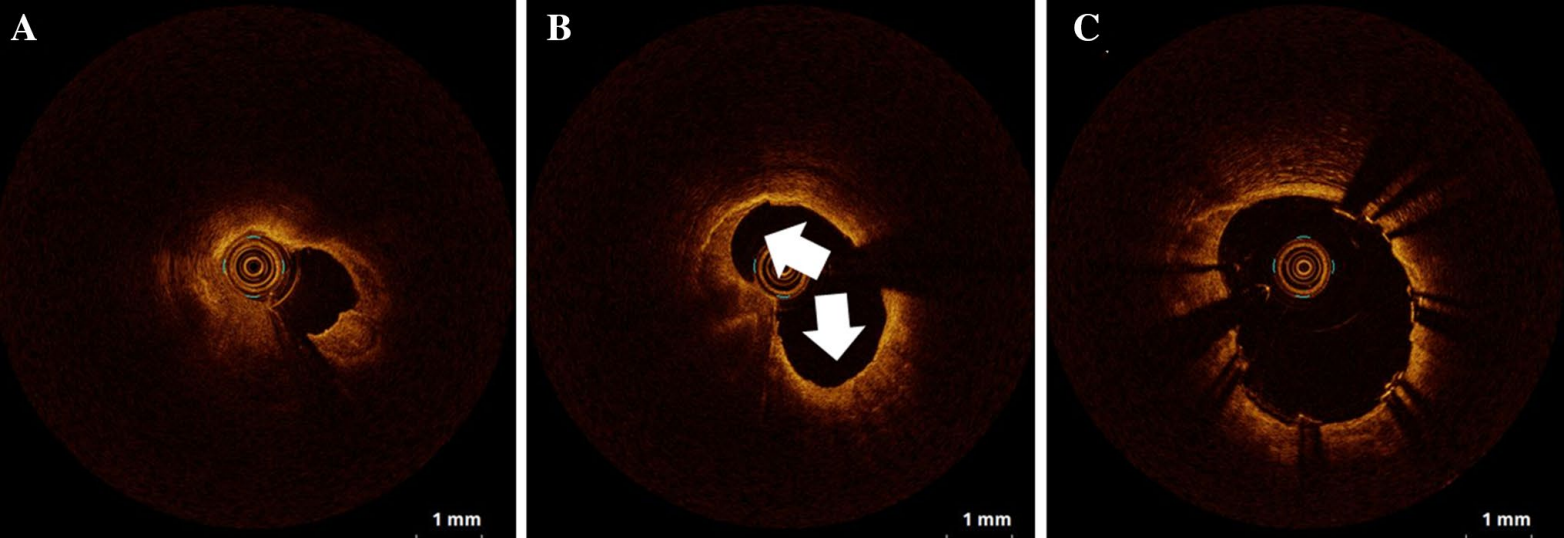
OCT-guided rotational atherectomy in severe calcified lesion. OCT image before rotational atherectomy reveals circumferential calcification (**a**). Calcified plaque ablated by rotational atherectomy is clearly visualized by OCT (**b**, *arrows*). After stent deployment, OCT can be used to make sure that the stent is well expanded

One of the greatest advantages of OCT is its high spatial resolution, and OCT can precisely measure lesion length and lumen diameter in PCI, which is useful in optimizing the size of dilation balloons and stents. The imaging procedure of intravascular OCT is similar to that of IVUS, except that blood must be displaced by saline or contrast medium while imaging. Since the OCT catheter has a short guide wire lumen at the distal portion of the catheter tip, the guide wire can be seen as a point artifact with shadowing, as in IVUS. Therefore, OCT can be used like IVUS, and it has been reported to alter the procedural strategy in over 80% of cases [26]. As mentioned above, OCT is the only intravascular imaging method that can accurately image calcium thickness without shadowing. It has been reported that the severity of calcification might be related to stent expansion [27]. Therefore, information on plaque components such as calcification is important to suggest the use of ancillary devices such as rotational atherectomy before stent implantation (Fig. 5) [28].

In terms of the comparison between angiography alone vs. angiography plus OCT-guided PCI, it has been reported that OCT-guided PCI had a significantly lower 1-year risk of cardiac death (1.2 vs. 4.5%, *p* = 0.010), cardiac death or myocardial infarction (6.6 vs. 13.0%, *p* = 0.006), and the composite of cardiac death, myocardial infarction, or repeat revascularization (9.6 vs. 14.8%, *p* = 0.044) [29]. This study was a case–control study and further large-scale prospective, randomized studies are needed to reveal the clinical superiority of OCT-guided PCI against angiography alone PCI. Regarding the comparison between OCT-guided and IVUS-guided PCI, Habara and colleagues first reported that OCT guidance for stent implantation was associated with smaller stent expansion than the conventional IVUS guidance in a single-center study. This result might be due to the poor visibility of the vessel border using OCT, which contributes to smaller stent expansion than with IVUS guidance. However, in a recent post hoc analysis, OCT and IVUS guidance for PCI resulted in a comparable degree of stent expansion [30]. More recently, in a randomized, controlled trial (ILUMIEN III: Optimize PCI trial), OCT-guided PCI using a specific reference segment external elastic lamina-based stent optimization strategy was safe and resulted in a similar degree of stent expansion to that of IVUS-guided PCI [31]. It has been reported that OFDI-guided PCI resulted in equivalent clinical and angiographic outcomes at 12 months in a multicenter, randomized, controlled trial (OPINION trial) [32]. Further large-scale prospective studies are needed to compare the outcomes between OCT-guided PCI and IVUS-guided PCI.

### Three-dimensional OCT

**Fig. 6:**
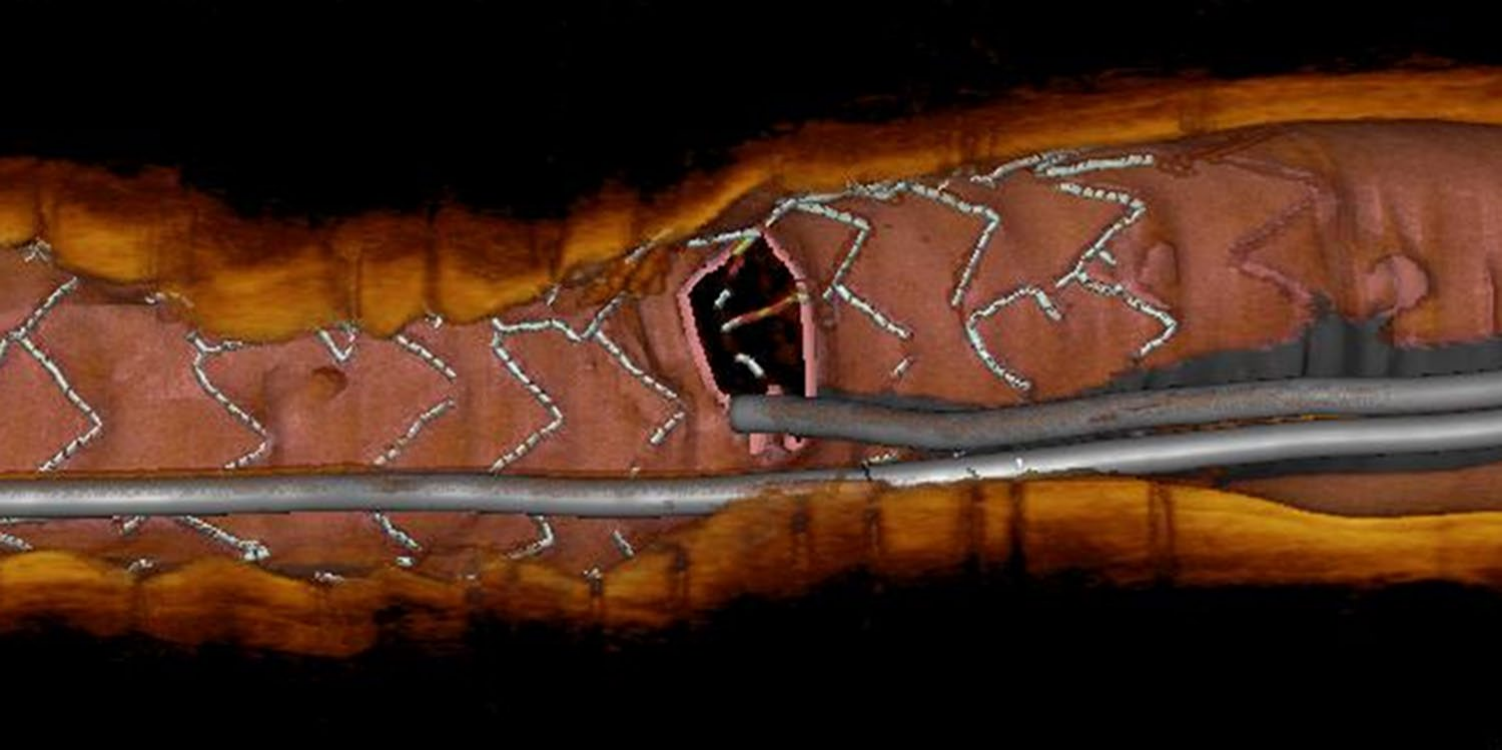
Three-dimensional OCT imaging in bifurcation PCI. 3D reconstruction with automatic stent strut detection can guide positioning of the wire through the appropriate stent cells to reduce the incidence of incomplete stent apposition in bifurcation lesions. The distal wiring position at the side-branch ostium could minimize stent deformity after side-branch balloon dilatation

The three-dimensional (3D) reconstruction of OCT was first described in 2008, and 3D OCT reconstruction technology has promptly shown its clinical utility [33]. 3D reconstruction with automatic stent strut detection is currently available in Japan, and this new technology can guide positioning of the wire through the appropriate stent cells to reduce the incidence of incomplete stent apposition in bifurcation lesions (Fig. 6) [34]. Furthermore, using 3D reconstruction OCT images with automatic stent accentuation, the side-branch orifice and the stent strut link location in bifurcation lesions treated with single-stent implantation are clearly visualized [35]. Previous pathological study demonstrated that high-intensity OCT signal tissues covering jailed struts at the side-branch orifice at follow-up contained smooth muscle cells with a proteoglycan–collagen matrix, suggesting normal neointimal tissue [36]. Therefore, normal neointimal coverage of struts at the side-branch orifice may play a protective role against future stent thrombosis. In contrast, neointimal coverage of struts at the side-branch orifice may be a cause of side-branch flow disturbance during long-term follow-up. Using 3D reconstruction OCT images, Nakamura et al. classified the configuration of overhanging struts at the side-branch orifice and compared the side-branch flow area (the area of the side-branch ostium except for jailing struts) among the no-jail type (N-type), the simple-jail type (S-type; no longitudinal link at the carina), and the complex-jail type (C-type; had a link at the carina) [37]. Percent reduction of side-branch flow area in the C-type group was significantly greater than that in the N-type or S-type groups during follow-up period. Stent jail complexity assessed by 3D reconstruction OCT might be associated with the progression of side-branch ostial stenosis.

### Co-registration of OCT with coronary angiography

**Fig. 7:**
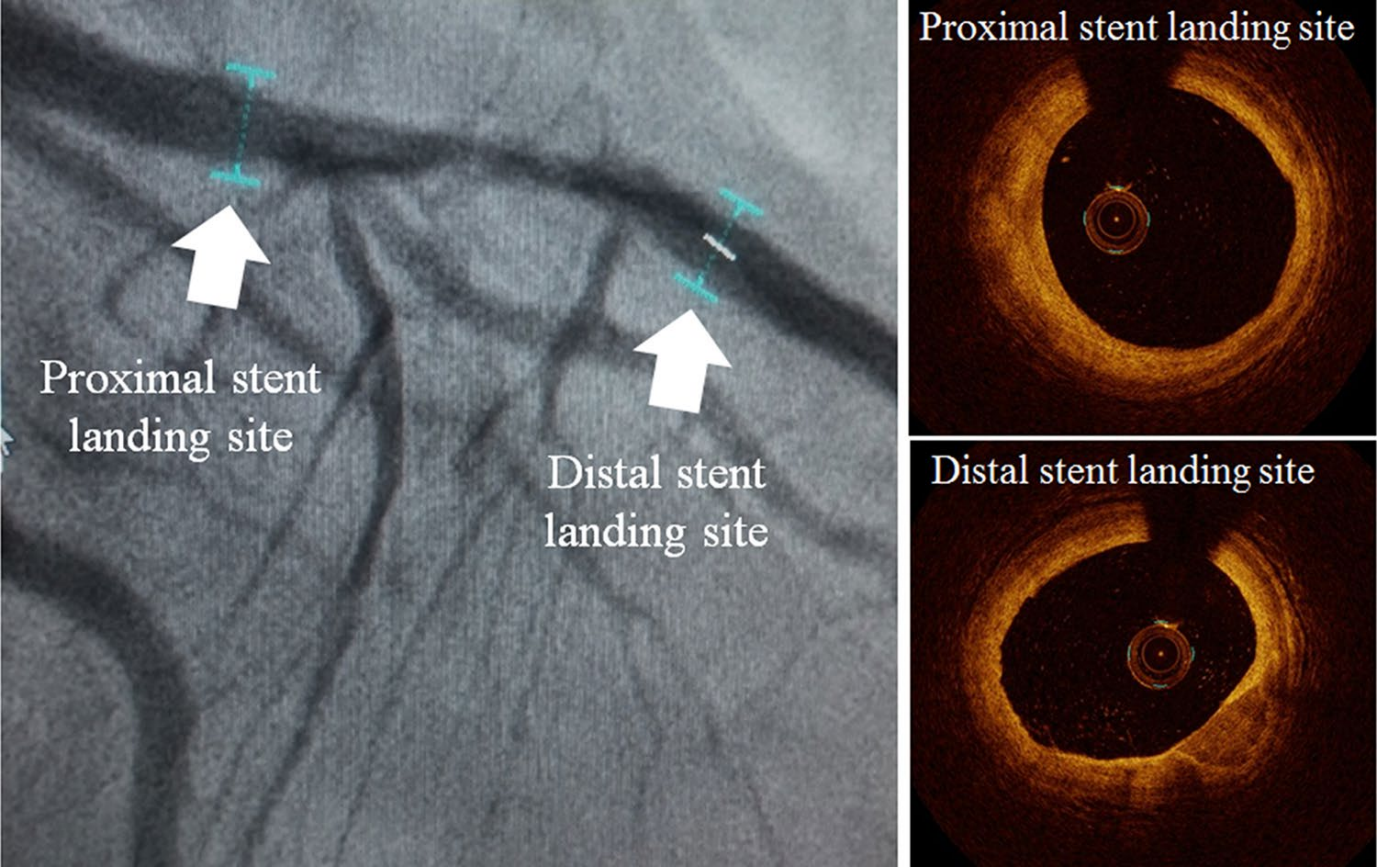
Stent roadmap with the co-registered angiography frames. The stent roadmap uses the OCT lumen profile measurements representing the locations of the appropriate distal and proximal “landing” sites for coronary stenting as markers within the co-registered angiography frames (OPTIS™ Metallic Stent Optimization Software, Abbott Vascular, Santa Clara, CA, USA)

**Fig. 8:**
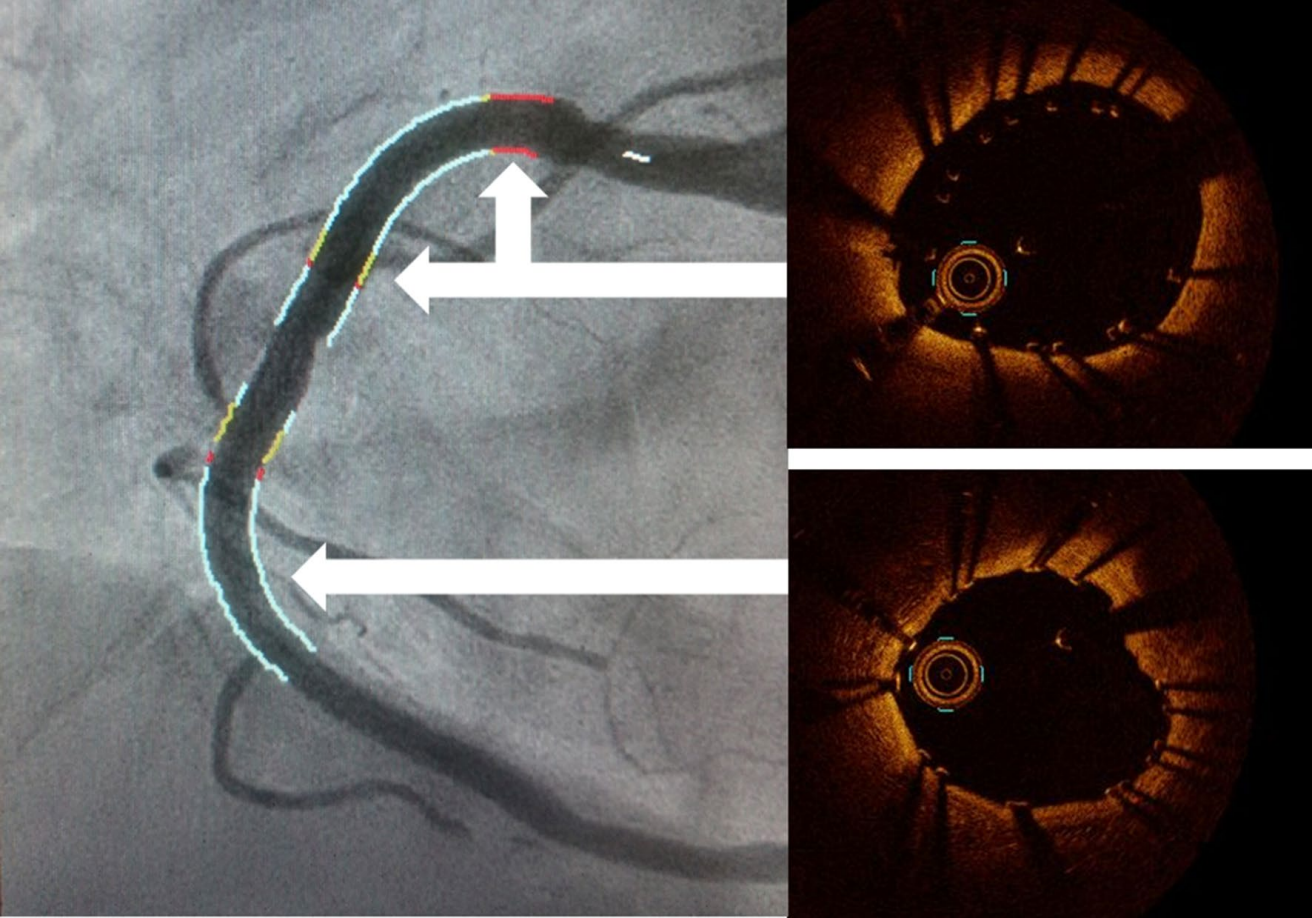
Stent apposition indicator with the co-registered angiography frames. After stent implantation, automatic stent strut detection enables a display apposition indicator with the co-registered angiography frames (*upper*; the *red* or *yellow line* indicates incomplete stent apposition, *lower*; the *silver line* indicates well-apposed stent apposition). This technology enables PCI operators to consider whether the stent is well-apposed to the coronary artery and determine if further stent expansion is needed. (OPTIS™ Metallic Stent Optimization Software, Abbott Vascular, Santa Clara, CA, USA)

A real-time point-to-point correspondence between coronary angiographic and OCT/OFDI images has been developed and provides useful information to PCI operators. The automatic point-to-point correspondence of OCT with coronary angiography enables the operator to map optimal stent implantation (Fig. 7). This software uses the OCT lumen profile measurements representing the locations of the appropriate distal and proximal “landing” sites for coronary stenting as markers within the co-registered angiography frames. This could reduce the risk of stent–vessel mismatch and an inappropriate “landing” site for stenting. Stent size selection with OCT is dictated by the smallest reference vessel diameter, which is usually the distal reference. In close-to-normal vessels, it is also dictated by the smallest reference lumen diameter. However, attention on the decrease in the luminal measurement of the reference sites is needed especially in lesions with tight stenosis. In terms of the distal reference site, the net change in lumen diameter was −0.12 mm, which is almost half of the size variation of most commercially available coronary stents [38]. In diseased vessels, we also pay attention to smaller stent expansion with OCT-guided PCI compared to IVUS-guided PCI due to the poor OCT visibility of the vessel border. As mentioned above in ILUMIEN III: Optimize PCI trial, with a standardized OCT strategy for stent sizing based on pre-PCI OCT measurements of external elastic lamina, to overcome the problem of incomplete vessel wall visualization, minimum stent area was comparable for OCT- as for IVUS-guided PCI (median 5.79 vs. 5.89 mm^2^, *p*_inferiority_ = 0.001) [31]. Stent sizing based on OCT measurements of external elastic lamina warrant a large-scale trial to establish whether or not OCT-guided PCI results in smaller stent expansion. The quality of the stent-landing zone may influence stent length selection. It has been widely known that interventional cardiologists prefer to position stents’ edges into normal or at least less diseases vessel references. Avoiding landing in eccentric calcified and lepidic plaques may prevent edge dissections [39] and stent edge restenosis [40]. Furthermore, after stent implantation, automatic stent strut detection enables evaluation of stent apposition and shows an apposition indicator with the co-registered angiography frames (Fig. 8). The red line on coronary angiogram indicates incomplete stent apposition, and the silver line indicates well-apposed stent apposition in OCT images. Although this technology has not been validated, yet it enables PCI operators to consider whether the stent is well-apposed to the coronary artery and determine if further stent expansion is needed on coronary angiogram.

## Conclusions

The unique capabilities of OCT as an investigational tool for high-risk lesions will serve the cardiology community well, as it moves us toward a better understanding of atherosclerotic plaque. In addition, because of the development of new OCT technology, OCT has become a notable catheter-based imaging technology that can provide practical guidance for PCI in clinical settings.
